# Open-Access Oesophagogastroduodenoscopy as an Effective and Safe Strategy for Patients With Non-alarming Symptoms

**DOI:** 10.7759/cureus.54792

**Published:** 2024-02-23

**Authors:** Yu Bin Tan, Chee Hooi Lim, Noor Azlina Binte Johari, Jason Pik Eu Chang, Malcolm Teck Kiang Tan

**Affiliations:** 1 Gastroenterology and Hepatology, Singapore General Hospital, Singapore, SGP

**Keywords:** open-access endoscopy, open-access oesophagogastroduodenoscopy, open-access gastroscopy, gastroduodenal disorders, upper gi endoscopy, dyspepsia, screening

## Abstract

Background: Open-access oesophagogastroduodenoscopy (OAO) is defined as the performance of oesophagogastroduodenoscopy (OGD) requested by referring physicians without a prior specialist consultation. With the increasing demand for specialist appointments, the use of OAO has helped to reduce healthcare utilization by decreasing prior clinic visits. This also allows endoscopies to be scheduled and performed earlier. This study aims to evaluate our experience in providing OAO services to patients with non-alarming dyspepsia symptoms under the age of 60.

Methods: The records of patients scheduled for OAO from January 2019 to December 2022 at Singapore General Hospital (SGH) Department of Gastroenterology were analyzed.

Results: Five hundred sixty-nine patients were scheduled for OAO, and 436 patients underwent the procedure. The mean age of patients was 45.7 (SD=10.9) years old. Thirty-six percent were males, and there were 80.8% Chinese, 5.3% Malay, 8.6% Indian, and 5.3% others. The median waiting time for endoscopy was 23 days (IQR 16-36), and no major adverse events were reported. Over half of the endoscopies were unremarkable (n=231, 53%). There were 25 (5.7%) patients with major findings; three had upper gastrointestinal adenocarcinoma (one oesophageal and two gastric), one had oesophageal varices, and 21 had peptic ulcer disease (10 gastric and 11 duodenal ulcers). A rapid urease test was conducted on 409 patients, and 55 (13.4%) were positive.

Conclusion: OAO is a safe and effective strategy for providing timely diagnostic OGD to normal-risk patients at our center. Primary care physicians are encouraged to refer non-alarming dyspepsia symptoms patients under 60 years for OAO over the conventional route.

## Introduction

Open-access oesophagogastroduodenoscopy (OAO) is defined as the performance of endoscopic procedures requested by referring physicians without a prior gastroenterology clinic consultation [1,2]. However, the increasing demand for oesophagogastroduodenoscopies (OGDs) has led to OAO being increasingly utilized worldwide, prompting the American Society for Gastrointestinal Endoscopy (ASGE) to update its guidelines in 2015 addressing this topic [3].

The concept of OAO was first introduced in the 1970s as a new approach to streamline the process of diagnostic endoscopy referral as a direct-to-treat care model without utilizing laboratory and radiological tests. The first paper published on this topic in 1979 described a three-year experience with an OAO service established at a district general hospital in England, with the conclusion that the significant increase in the number of endoscopies could not be justified as there was “too little objective benefit” [4].

While there is no official data available on the OGD utilization rate in Singapore, a report published by the Saw Swee Hock School of Public Health analyzed the trends in OGD utilization from 2014 to 2019, showing rising rates of OGD claims increasing twofold from 8.9 per 1000 insurance policyholders in 2014 to 17.3 per 1000 policyholders in 2019 [5]. This mirrors global trends, and proponents of OAO suggest that OAO would reduce overall costs and waiting time at the clinic by eliminating clinic consultations prior to endoscopy and allowing timely OGDs to be performed [6].

A national gastroenterology working group was formed in Singapore in 2014 to undertake a fundamental review of the specialist outpatient clinic (SOC) service delivery model referred from subsidized primary care. As a result, the OAO service was first introduced in Singapore at the National Healthcare Group and National University Health System hospitals in 2015. SGH started a pilot OAO service in 2017. This service is available in all public hospitals as of 2019.

The primary aim of this study is to show that OAO is safe and that a prior clinic visit to a gastroenterologist is not needed for selected patients with non-alarming dyspepsia symptoms under the age of 60. The secondary objectives are to describe the epidemiology and findings of patients who underwent OAO, the time to OAO, and to also show that successful OAO leads to low re-referral rates.

This is the first local study from Singapore to look at the outcomes of the OAO program at the SGH over a four-year period.

## Materials and methods

This is a retrospective analysis. The records of patients scheduled for OAO from January 2019 to December 2022 at the SGH Department of Gastroenterology and Hepatology were reviewed and analyzed. Systemic collection was prospectively recorded as part of quality assurance measures. The Singapore General Hospital approved the collection of data for this study (SGH-2023-02-00079).

Patients and OAO service

Polyclinics (health facilities with primary care physicians) across Singapore can refer patients to any publicly restructured hospital. For appropriate patients, they are encouraged to refer to OAO as an alternative to the traditional system of referral to SOC. The waiting time for an appointment from a polyclinic referral to our SOC ranges from two weeks to four months, with a median waiting time of three to four months post-pandemic (unpublished data). OAO appointments are arranged, and the procedure is then scheduled to be done within six weeks of referral. SOC follow-up is arranged for patients when clinically indicated.

The inclusion criteria for OAO included patients between the ages of 21 and 60 with non-alarming dyspepsia symptoms such as reflux, heartburn, recurrent abdominal pain, and bloating. Patients at potentially higher risk of complications from sedation (severe ischemic heart disease/cardiac devices/heart valve replacements, severe pulmonary disease, poorly controlled hypertension or diabetes, acute coronary syndrome or stroke within the last six months, difficult airway issues, anticoagulation users) or those that require urgent endoscopy (signs of gastrointestinal bleeding) were excluded from utilizing OAO service (Table 1).

**Table 1:** Inclusion and exclusion criteria for OAO at SGH OAO: open-access oesophagogastroduodenoscopy, SGH: Singapore General Hospital, BP: blood pressure, NOAC: non-vitamin K antagonist oral anticoagulant

Inclusion	Exclusion
Age 21-60 years old	Physically unfit
Non-alarming dyspepsia symptoms: reflux, heartburn, recurrent abdominal pain, bloating	Uncontrolled hypertension (BP >180/100)
	Diabetic on insulin
	Severe ischaemic heart disease/with cardiac device, e.g., cardiac pacemakers and stents/heart valve replacements
	Severe pulmonary disease
	Below 21 years or above 60 years
	Pregnancy
	Hematemesis or melaena (consider emergency department referral)
	Ongoing fresh PR bleeding (consider emergency department referral)
	On warfarin and NOAC medications (e.g., dabigatran, rivaroxaban, apixaban, edoxaban)
	Not competent to give consent
	Significant loss of weight
	Acute coronary syndrome/cerebrovascular accident within six months
	Difficult airway (e.g., short chin, obstructive sleep apnea, morbid obesity)

Evaluations

Demographic data, including age, gender, and ethnicity, were recorded. The scheduled time to scope is the period between the date of request for OAO and the first appointment date for OAO. The actual time to scope is the period between the date of request for OAO and the date patients had their OAO. The OGD diagnostic outcomes were analyzed. We defined major findings as findings that require an early intervention or referral. Examples include oesophageal or gastric neoplasia, varix, or peptic ulcer disease. Minor findings include oesophagitis, gastritis, duodenitis, hiatus hernia, and benign polyps. We recorded post-OGD adverse events that were defined as endoscopic complications such as bleeding, perforation, peri-procedure or post-procedure hemodynamic instability, a cardiac event, or any event leading to unscheduled admission within eight days (one week after OAO) [7]. Descriptive statistics include means with standard deviations and medians with interquartile ranges, depending on the distribution of data.

## Results

A total of 567 patients were scheduled for OAO, and 436 patients underwent the procedure. The mean age of patients was 45.7 (SD=10.9) years old, 36.3% were males, and there were 80.8% Chinese, 5.3% Malay, 8.6% Indian, and 5.3% others (Table 2).

**Table 2:** Patient demographics

Demographics	
Mean age (SD)	45.7 (10.9) years old
Gender	
Male (%)	206 (36.3)
Female (%)	361 (63.7)
Race	
Chinese (%)	458 (80.8)
Malay (%)	30 (5.3)
Indian (%)	49 (8.6)
Others (%)	30 (5.3)

The median waiting time for endoscopy was 23 days (IQR 16-36). This is similar to the actual waiting time for endoscopy and highlights that there are no frequent cancellations or postponements to OAO dates. There were also no major adverse events reported. Over half of the endoscopies were unremarkable (231, 53.0%). There were 25 (5.7%) patients with major findings; three had upper gastrointestinal adenocarcinoma (one oesophageal and two gastric), one had incidental oesophageal varices and 21 had peptic ulcer disease (10 gastric and 11 duodenal ulcers). One hundred eighty (41.3%) patients had minor findings, which included gastritis/oesophagitis, gastric polyps, and hiatal hernia (Table 3).

**Table 3:** Performance and clinical outcomes OAO: open-access oesophagogastroduodenoscopy, COVID-19: coronavirus disease 2019, IQR: interquartile range

Outcome measures and findings	
Scheduled OAO	567
Actualized OAO	436
No shows	91 (16.0%)
Cancellations	40 (7.1%)
Cancellations related to COVID-19	11
Median scheduled time to scope (IQR)	23 (17-30) days
Median actual time to scope (IQR)	23 (16-36) days
Normal findings	231 (53.0%)
Major findings	25 (5.7%)
Gastric/oesophageal adenocarcinoma	3
Varices	1
Peptic ulcer disease	21 (10 gastric and 11 duodenal ulcers)
Minor findings	180 (41.3%)
Gastritis/duodenitis	72
Oesophagitis	45
Hiatal hernia	32
Polyps	26
Others	5
Rapid urease test performed	409
Rapid urease test positive	55 (13.4%)
Rapid urease test negative	354 (86.6%)
Adverse events	0
Gastroenterology follow-up appointment	85 (19.5%)
Re-referral to SOC 1 year after scheduled OAO	17 (2.99%)
Re-referral to SOC 1 year after actual OAO	16 (3.65%)

A rapid urease test was conducted on 409 patients, and 55 (13.4%) returned positive. A majority of patients (80.5%) were discharged back to their primary care physicians after their OGD was performed. The no-show rate was 91 (16%), while the cancellation rate, where patients called in to cancel the procedure, was 7.4%. Eleven (26.2%) patients who canceled cited COVID-19 as the reason for the cancellation.

The re-referral rate back to SOC for dyspepsia symptoms from primary care physicians within a year after OAO was 16 patients (3.7%). If no shows or cancellations were included, the re-referral rate within a year was 17 patients (3.0%). Fifteen patients, representing only 2.6% of the total patients referred, were rejected over the last four years as they did not meet the inclusion and exclusion criteria for OAO.

## Discussion

OGD is a common and safe procedure with minimal preparation needed, making it an ideal procedure for an open-access program. However, several issues have arisen as a result of the implementation of OAO worldwide. A commonly raised concern is the appropriateness of endoscopy, which is relevant as the referrals are often made by primary care physicians who may not be familiar with the indications of the procedure. Multiple studies have shown that inappropriate referrals range from 5% to 49%, and the ASGE guideline on “appropriate use of GI endoscopy” was commonly used as a reference to determine if the referral was appropriate [8-16]. This issue needs to be addressed as inappropriate referrals may lead to canceled procedures or unnecessary risks and costs to patients and the healthcare system, thus defeating the purpose of streamlining the endoscopy process both in timeliness and cost-effectiveness. In our OAO program, only 2.6% of our patients did not meet the inclusion criteria and were deemed inappropriate referrals. This is lower than previous studies in the literature and could be due to a lower age inclusion criteria of 21 years old, as younger patients tend to have non-alarming symptoms. In addition, we have a checklist of inclusion and exclusion criteria in the referring form to minimize inappropriate OAO referrals.

Several studies have also shown that appropriate OAO referrals lead to a higher diagnostic yield of clinically relevant findings. One large prospective multicenter Italian study of 6270 patients noted a significantly higher diagnostic yield for appropriate OGDs compared to those that were deemed inappropriate (52% vs. 29%) [13]. In our study, 47% of the OAO had findings, with 5.7% having major findings such as a malignancy or ulcer and another 41.3% having minor findings. The number of cancer diagnoses in our OAO cohort is similar to that of the Hong Kong OAO service. Their OAO model is also comparable to ours, i.e., primary care is free to choose OAO or traditional clinic referral service [6]. This suggests that OAO programs that have high adherence to appropriate indications would be able to achieve good diagnostic yield, and this metric should be considered an appropriate quality indicator for OAO programs worldwide.

We found the prevalence of *Helicobacter pylori* detected on a rapid urease test to be low, at 13.4%. Previously, the seroprevalence of *Helicobacter pylori* in Singapore was estimated at 31.4% [17]. However, this data was obtained from healthy individuals who were blood donors and from hospitalized pediatric patients without gastrointestinal diseases. Our study is thus the first endoscopic study showing the prevalence of adult patients with non-alarming dyspepsia symptoms and *Helicobacter pylori* in Singapore. However, as many of these patients may already be on proton pump inhibitors, this value may be falsely low due to the increased false negative rates of the rapid urease test on these agents. Another limitation is that the rapid urease test was not performed on all patients and was only performed at the discretion of the endoscopist.

Notably, the follow-up rate post-OGD in our program was 19.5%, as our workflow requires SOC follow-up for any outstanding histology results. This was higher than the 7% that was found in a retrospective study published by Charles et al. on 168 patients who had open-access endoscopy at their center [12]. However, as the study also included patients who underwent open-access colonoscopy, the data may not be comparable. We are also reviewing an alternative process of communicating benign histology results to patients, thus avoiding a SOC follow-up.

No-show rates are an important factor in healthcare resource utilization and have been estimated to be between 12 and 27% globally. Various reasons have been cited for no-shows, such as illness on the day of the procedure, anxiety, symptom resolution, and mistaking/forgetting appointment dates and times [18-22]. Separately, the ASGE GI Operations Benchmarking Survey 2019 Databook reported that cancellation rates range from 5.6% to 8.5% [23]. While no data is publicly available on the no-show and cancellation rates in Singapore, our study had a no-show rate of 16% and a cancellation rate of 7.4%, which was slightly lower than the no-show rate of 24% in previous data from Asian literature [6]. This was despite the COVID-19 pandemic. While the estimated cost of these no-shows and cancellations is not known, a study conducted by Berg et al. in the USA estimated that the daily loss attributed to a no-show rate of 18% is USD$725.42 based on a simulation model that was designed in their study [24].

Importantly, no adverse events occurred, thus suggesting that the safety of our patients was not compromised despite the absence of an initial specialist consultation prior to endoscopy. Our study showed a low re-referral rate of 3.7% back to the Gastroenterology SOC for dyspepsia symptoms within a year after OAO was performed. Most re-referrals were due to recurrent symptoms despite an OGD and where the primary care physician felt a specialist input would be appropriate. While there is a lack of data in the literature for comparison, we believe that this rate is sufficiently low, thus justifying the effectiveness of OAO for patients with dyspepsia and non-alarming symptoms.

There are several limitations to this study. The first limitation is that our cohort is from a single OAO center. Hence, we are unable to analyze the utilization data for OAO service, which we define as the percentage of patients fulfilling the referral criteria who are actually referred to OAO service. The second limitation is the lack of a control group, which would have allowed us to compare the wait time and cost-effectiveness of the OAO program. However, we do know that the waiting time for an appointment from a polyclinic referral to our SOC ranges from two weeks to four months, with a median waiting time of three to four months post-pandemic (unpublished data). Our OAO program showed that the median wait time for an OAO is 23 days. This shows that even without the control group, OAO is more cost-effective and timely compared to the traditional system of referral to our SOC, as it is faster and also saves a visit.

## Conclusions

OAO is a safe and effective service for those under 60 years old with non-alarming dyspepsia symptoms. We are able to maintain referrals to OAO appointments under six weeks, providing an alternative, timely service for this group of patients. We advocate OAO service and recommend the implementation of science studies to increase the utilization rate.
